# Supplementary material for: Time-series transcriptome analysis identified differentially expressed genes in broiler chicken infected with mixed Eimeria species
Source: Front Genet. 2022 Aug 8;13:886781. doi: 10.3389/fgene.2022.886781 (PMC9393255; doi:10.3389/fgene.2022.886781)
Supplement: Supplementary file 2 [file DataSheet1.ZIP › 7dpi_GO.Gsea.1625106495923/GOCC_RIBOSOMAL_SUBUNIT.html]

Details for gene set GOCC\_RIBOSOMAL\_SUBUNIT[GSEA]

|  || Dataset | TMM\_7dpi\_gct\_format\_7dpi\_gct\_format.Class\_4dpi.cls #PC\_versus\_NC.Class\_4dpi.cls #PC\_versus\_NC\_repos |
| Phenotype | Class\_4dpi.cls#PC\_versus\_NC\_repos |
| Upregulated in class | 1 |
| GeneSet | GOCC\_RIBOSOMAL\_SUBUNIT |
| Enrichment Score (ES) | 0.57612705 |
| Normalized Enrichment Score (NES) | 3.0595834 |
| Nominal p-value | 0.0 |
| FDR q-value | 0.0 |
| FWER p-Value | 0.0 |
Table: GSEA Results Summary

  

Fig 1: Enrichment plot: GOCC\_RIBOSOMAL\_SUBUNIT      
 Profile of the Running ES Score & Positions of GeneSet Members on the Rank Ordered List

  

| SYMBOL | TITLE | RANK IN GENE LIST | RANK METRIC SCORE | RUNNING ES | CORE ENRICHMENT || 1 | MRPS12 | na | 242 | 0.999 | 0.0025 | Yes |
| 2 | MRPS6 | na | 315 | 0.928 | 0.0178 | Yes |
| 3 | MRPL30 | na | 351 | 0.883 | 0.0351 | Yes |
| 4 | NDUFAB1 | na | 356 | 0.876 | 0.0549 | Yes |
| 5 | MRPL43 | na | 504 | 0.741 | 0.0596 | Yes |
| 6 | RPS28 | na | 529 | 0.721 | 0.0741 | Yes |
| 7 | MRPL23 | na | 564 | 0.704 | 0.0874 | Yes |
| 8 | MRPL12 | na | 579 | 0.694 | 0.1022 | Yes |
| 9 | MRPL57 | na | 643 | 0.665 | 0.1122 | Yes |
| 10 | MPV17L2 | na | 697 | 0.638 | 0.1224 | Yes |
| 11 | RPS29 | na | 740 | 0.617 | 0.1330 | Yes |
| 12 | MRPL58 | na | 755 | 0.609 | 0.1458 | Yes |
| 13 | MRPL54 | na | 779 | 0.596 | 0.1576 | Yes |
| 14 | MRPS33 | na | 798 | 0.591 | 0.1697 | Yes |
| 15 | RPS27L | na | 833 | 0.579 | 0.1801 | Yes |
| 16 | MRPL35 | na | 853 | 0.571 | 0.1916 | Yes |
| 17 | MRPL10 | na | 872 | 0.563 | 0.2031 | Yes |
| 18 | MRPL55 | na | 880 | 0.561 | 0.2154 | Yes |
| 19 | MRPS16 | na | 950 | 0.538 | 0.2219 | Yes |
| 20 | RPL26L1 | na | 1025 | 0.512 | 0.2274 | Yes |
| 21 | MRPL21 | na | 1141 | 0.472 | 0.2286 | Yes |
| 22 | MRPL15 | na | 1217 | 0.450 | 0.2326 | Yes |
| 23 | MRPS10 | na | 1264 | 0.437 | 0.2388 | Yes |
| 24 | MRPS28 | na | 1280 | 0.432 | 0.2475 | Yes |
| 25 | MRPL40 | na | 1282 | 0.431 | 0.2573 | Yes |
| 26 | MRPL45 | na | 1312 | 0.427 | 0.2647 | Yes |
| 27 | UBA52 | na | 1369 | 0.413 | 0.2694 | Yes |
| 28 | MRPL46 | na | 1380 | 0.409 | 0.2780 | Yes |
| 29 | MRPL28 | na | 1382 | 0.409 | 0.2873 | Yes |
| 30 | NSUN4 | na | 1403 | 0.404 | 0.2949 | Yes |
| 31 | RPS27 | na | 1424 | 0.399 | 0.3024 | Yes |
| 32 | MRPL9 | na | 1437 | 0.396 | 0.3105 | Yes |
| 33 | MRPL3 | na | 1475 | 0.387 | 0.3162 | Yes |
| 34 | MRPL42 | na | 1521 | 0.375 | 0.3211 | Yes |
| 35 | MRPL19 | na | 1535 | 0.370 | 0.3285 | Yes |
| 36 | RPS19 | na | 1540 | 0.368 | 0.3366 | Yes |
| 37 | MRPL47 | na | 1550 | 0.367 | 0.3443 | Yes |
| 38 | RPL38 | na | 1576 | 0.361 | 0.3505 | Yes |
| 39 | RPL36 | na | 1577 | 0.361 | 0.3587 | Yes |
| 40 | MRPL1 | na | 1639 | 0.348 | 0.3616 | Yes |
| 41 | MRPS17 | na | 1651 | 0.345 | 0.3686 | Yes |
| 42 | MRPL34 | na | 1672 | 0.339 | 0.3747 | Yes |
| 43 | MRPS35 | na | 1686 | 0.336 | 0.3813 | Yes |
| 44 | MRPS11 | na | 1692 | 0.335 | 0.3886 | Yes |
| 45 | MRPL33 | na | 1696 | 0.333 | 0.3960 | Yes |
| 46 | MRPS18C | na | 1729 | 0.325 | 0.4008 | Yes |
| 47 | MRPL37 | na | 1736 | 0.324 | 0.4077 | Yes |
| 48 | RPL23A | na | 1779 | 0.313 | 0.4114 | Yes |
| 49 | RPL29 | na | 1780 | 0.313 | 0.4185 | Yes |
| 50 | MRPL44 | na | 1799 | 0.307 | 0.4241 | Yes |
| 51 | RPS15 | na | 1804 | 0.306 | 0.4308 | Yes |
| 52 | RPLP1 | na | 1813 | 0.304 | 0.4371 | Yes |
| 53 | RPL36A | na | 1833 | 0.301 | 0.4424 | Yes |
| 54 | RPL19 | na | 1834 | 0.300 | 0.4493 | Yes |
| 55 | MRPL2 | na | 1843 | 0.299 | 0.4555 | Yes |
| 56 | MRPS21 | na | 1864 | 0.296 | 0.4606 | Yes |
| 57 | MRPL16 | na | 1884 | 0.291 | 0.4657 | Yes |
| 58 | RPL39L | na | 1966 | 0.277 | 0.4652 | Yes |
| 59 | RPL37A | na | 1983 | 0.274 | 0.4702 | Yes |
| 60 | MRPL53 | na | 1984 | 0.273 | 0.4764 | Yes |
| 61 | MRPL41 | na | 2082 | 0.257 | 0.4742 | Yes |
| 62 | MRPL24 | na | 2093 | 0.254 | 0.4792 | Yes |
| 63 | MRPL13 | na | 2109 | 0.251 | 0.4837 | Yes |
| 64 | MRPS9 | na | 2193 | 0.238 | 0.4821 | Yes |
| 65 | RPL22 | na | 2201 | 0.237 | 0.4870 | Yes |
| 66 | MRPL48 | na | 2228 | 0.232 | 0.4901 | Yes |
| 67 | RPL35A | na | 2241 | 0.231 | 0.4944 | Yes |
| 68 | RSL24D1 | na | 2281 | 0.225 | 0.4963 | Yes |
| 69 | MRPL14 | na | 2284 | 0.224 | 0.5013 | Yes |
| 70 | RPL34 | na | 2286 | 0.224 | 0.5064 | Yes |
| 71 | RPL27 | na | 2300 | 0.222 | 0.5104 | Yes |
| 72 | MRPS14 | na | 2305 | 0.222 | 0.5151 | Yes |
| 73 | RPS26 | na | 2332 | 0.216 | 0.5179 | Yes |
| 74 | RPL14 | na | 2343 | 0.215 | 0.5220 | Yes |
| 75 | RPS24 | na | 2347 | 0.214 | 0.5267 | Yes |
| 76 | MRPS18A | na | 2363 | 0.213 | 0.5303 | Yes |
| 77 | RPL23 | na | 2383 | 0.209 | 0.5335 | Yes |
| 78 | RPL7L1 | na | 2403 | 0.207 | 0.5367 | Yes |
| 79 | RPS7 | na | 2413 | 0.204 | 0.5406 | Yes |
| 80 | MRPS34 | na | 2496 | 0.193 | 0.5381 | Yes |
| 81 | MRPL18 | na | 2512 | 0.189 | 0.5412 | Yes |
| 82 | MRPS5 | na | 2649 | 0.170 | 0.5336 | Yes |
| 83 | RPL7 | na | 2670 | 0.168 | 0.5358 | Yes |
| 84 | MRPL39 | na | 2682 | 0.166 | 0.5387 | Yes |
| 85 | HBA1 | na | 2688 | 0.165 | 0.5421 | Yes |
| 86 | RPL31 | na | 2693 | 0.164 | 0.5455 | Yes |
| 87 | RPL24 | na | 2734 | 0.160 | 0.5458 | Yes |
| 88 | MRPL22 | na | 2747 | 0.158 | 0.5484 | Yes |
| 89 | MCTS1 | na | 2752 | 0.157 | 0.5517 | Yes |
| 90 | RPS8 | na | 2771 | 0.154 | 0.5537 | Yes |
| 91 | MRPL20 | na | 2783 | 0.153 | 0.5563 | Yes |
| 92 | RPL21 | na | 2810 | 0.149 | 0.5575 | Yes |
| 93 | RPL32 | na | 2870 | 0.142 | 0.5558 | Yes |
| 94 | EIF2A | na | 2889 | 0.139 | 0.5575 | Yes |
| 95 | RPL13 | na | 2902 | 0.138 | 0.5597 | Yes |
| 96 | MRPL38 | na | 2918 | 0.137 | 0.5615 | Yes |
| 97 | RPS17 | na | 2988 | 0.128 | 0.5586 | Yes |
| 98 | RPS15A | na | 3019 | 0.124 | 0.5590 | Yes |
| 99 | RACK1 | na | 3023 | 0.123 | 0.5616 | Yes |
| 100 | RPS13 | na | 3028 | 0.122 | 0.5640 | Yes |
| 101 | DAP3 | na | 3039 | 0.121 | 0.5660 | Yes |
| 102 | RPLP2 | na | 3040 | 0.121 | 0.5687 | Yes |
| 103 | RPL6 | na | 3048 | 0.121 | 0.5709 | Yes |
| 104 | RPS14 | na | 3050 | 0.120 | 0.5736 | Yes |
| 105 | RPL27A | na | 3078 | 0.116 | 0.5740 | Yes |
| 106 | RPS20 | na | 3085 | 0.115 | 0.5761 | Yes |
| 107 | RPL9 | na | 3146 | 0.107 | 0.5735 | No |
| 108 | MRPL27 | na | 3152 | 0.107 | 0.5756 | No |
| 109 | MRPS7 | na | 3210 | 0.100 | 0.5731 | No |
| 110 | MRPL51 | na | 3225 | 0.098 | 0.5741 | No |
| 111 | MRPS22 | na | 3240 | 0.095 | 0.5751 | No |
| 112 | RPS25 | na | 3324 | 0.084 | 0.5701 | No |
| 113 | RPL30 | na | 3365 | 0.078 | 0.5685 | No |
| 114 | RPS10 | na | 3396 | 0.074 | 0.5677 | No |
| 115 | RPL11 | na | 3414 | 0.072 | 0.5679 | No |
| 116 | MRPS31 | na | 3424 | 0.071 | 0.5688 | No |
| 117 | MRPS26 | na | 3453 | 0.068 | 0.5680 | No |
| 118 | RPL7A | na | 3462 | 0.067 | 0.5688 | No |
| 119 | RPL35 | na | 3487 | 0.064 | 0.5683 | No |
| 120 | RPS21 | na | 3592 | 0.053 | 0.5607 | No |
| 121 | RPS16 | na | 3626 | 0.051 | 0.5591 | No |
| 122 | RPL18A | na | 3668 | 0.045 | 0.5567 | No |
| 123 | RPS11 | na | 3727 | 0.037 | 0.5526 | No |
| 124 | RPS2 | na | 3817 | 0.025 | 0.5457 | No |
| 125 | MRPL32 | na | 3822 | 0.025 | 0.5460 | No |
| 126 | RPL10A | na | 3828 | 0.024 | 0.5461 | No |
| 127 | MRPS27 | na | 3855 | 0.021 | 0.5444 | No |
| 128 | RPL3 | na | 3883 | 0.018 | 0.5425 | No |
| 129 | RPL37 | na | 4179 | -0.015 | 0.5180 | No |
| 130 | RPS27A | na | 4189 | -0.016 | 0.5176 | No |
| 131 | RPL8 | na | 4274 | -0.027 | 0.5111 | No |
| 132 | RPS12 | na | 4369 | -0.037 | 0.5040 | No |
| 133 | RPS3A | na | 4499 | -0.050 | 0.4943 | No |
| 134 | RPL5 | na | 4509 | -0.051 | 0.4947 | No |
| 135 | MRPS2 | na | 4677 | -0.068 | 0.4822 | No |
| 136 | MALSU1 | na | 4809 | -0.082 | 0.4730 | No |
| 137 | RPL15 | na | 4832 | -0.084 | 0.4731 | No |
| 138 | RPL12 | na | 4912 | -0.094 | 0.4686 | No |
| 139 | LARP4 | na | 5084 | -0.111 | 0.4567 | No |
| 140 | RPL4 | na | 5112 | -0.114 | 0.4571 | No |
| 141 | RPLP0 | na | 5368 | -0.137 | 0.4387 | No |
| 142 | RPS4Y1 | na | 5409 | -0.140 | 0.4386 | No |
| 143 | RBM3 | na | 5497 | -0.149 | 0.4346 | No |
| 144 | RPS3 | na | 5595 | -0.158 | 0.4301 | No |
| 145 | MRPS36 | na | 5609 | -0.160 | 0.4327 | No |
| 146 | MRPL17 | na | 5768 | -0.177 | 0.4234 | No |
| 147 | EIF2D | na | 6138 | -0.218 | 0.3973 | No |
| 148 | MRPL50 | na | 6658 | -0.271 | 0.3597 | No |
| 149 | RPL17 | na | 7417 | -0.353 | 0.3039 | No |
| 150 | RPS23 | na | 7461 | -0.357 | 0.3085 | No |
| 151 | RPS6 | na | 7644 | -0.376 | 0.3018 | No |
| 152 | MRPS30 | na | 8183 | -0.444 | 0.2666 | No |
| 153 | DDX3X | na | 9281 | -0.596 | 0.1878 | No |
| 154 | NSUN3 | na | 10405 | -0.791 | 0.1112 | No |
| 155 | DHX29 | na | 11322 | -1.046 | 0.0580 | No |
Table: GSEA details [plain text format]

  

Fig 2: GOCC\_RIBOSOMAL\_SUBUNIT      
 Blue-Pink O' Gram in the Space of the Analyzed GeneSet

  

Fig 3: GOCC\_RIBOSOMAL\_SUBUNIT: Random ES distribution      
 Gene set null distribution of ES for **GOCC\_RIBOSOMAL\_SUBUNIT**
